# Use of a commercial ion chamber detector array for the measurement of high spatial‐resolution photon beam profiles

**DOI:** 10.1002/acm2.12466

**Published:** 2018-10-04

**Authors:** Vida Karimnia, Matthew D. Belley, Robert Rodgers, Michael Price

**Affiliations:** ^1^ Department of Physics University of Rhode Island Kingston RI USA; ^2^ Lifespan Cancer Institute Providence RI USA; ^3^ Warren Alpert Medical School of Brown University Providence RI USA

**Keywords:** gamma analysis, ion chamber array, photon beam profiles, quality assurance

## Abstract

Linear accelerator (linac) commissioning and quality assurance measurements are time‐consuming tasks that often require a water tank scanning system to acquire profile scans for full characterization of dosimetric beam properties. To increase efficiency, a method is demonstrated to acquire variable resolution, photon beam profile data using a commercially available ion chamber array (0.5 cm detector spacing). Field sizes of 2 × 2, 5 × 5, 10 × 10, and 15 × 15 cm^2^ were acquired at depths in solid water of *d*_max_, 5 cm, and 10 cm; additionally, beam profiles for field sizes of 25 × 25 and 40 × 40 cm^2^ were acquired at 5 cm depth in solid water at x‐ray energies of 6 and 23 MV. 1D composite profiles were generated by combining discrete point measurements made at multiple couch positions. The 1D composite profile dataset was evaluated against a commissioning dataset acquired with a 3D water tank scan system utilizing (a) 0.125 cc ion chamber for 5 × 5, 10 × 10, 15 × 15, 25 × 25, and 40 × 40 field sizes and (b) a solid state detector for 2 × 2 cm^2^ field size. The two datasets were compared to the gamma criteria at 1%/1 mm and 2%/2 mm tolerance. Almost all pass rates exceeded 95% at 2%/2 mm except for the 6 MV 2 × 2 cm^2^ field size at *d*_max_. Pass rates at 1%/1 mm ranged from 51% to 99%, with an average pass rate of 82%. A fourfold reduction in MU was achieved for scans larger than 15 × 15 cm^2^ using this method compared to the water tank scans. Further, dynamic wedge measurements acquired with the ion chamber array showed reasonable agreement with the treatment planning system. This method opens up new possibilities for rapid acquisition of variable resolution 2D–3D dosimetric data mitigating the need for acquiring all scan data with in‐water measurements.

## 1 INTRODUCTION

Linear accelerator (linac) commissioning includes a series of measurements by medical physicists to benchmark beam characteristics, linac performance, and functionality. Data collected at commissioning are used to model the treatment planning system and to define quality assurance baselines. This task is important since it directly relates to the quality of future patient treatments. Intensity modulated radiation therapy (IMRT), volumetric modulated arc therapy (VMAT), and stereotactic radiotherapy (SRT) are among modern treatment modalities that need accurate commissioning of beam data.^1^, ^2^, ^3^ Wide use of these technologies suggests that inaccurate beam models in the treatment planning system used to optimize and plan the linac delivery has the potential to do widespread harm. High quality data must be acquired at the time of commissioning and verified annually to ensure that the dose delivered by the linac matches the model in the planning software.

Two categories of data collected during commissioning are scan data and non‐scan data. Scan data require translation of a radiation sensor through the beam to measure percent depth dose (PDD), inline, and crossline profiles at different depths for open and wedge fields that are common for photon and electron beams. Non‐scan data include static point measurements, often normalized relative to a reference condition, as needed to measure inter‐ and intra‐leaf leakages for the multileaf collimator (MLC), scatter factors, tray and wedge factors, cone factors, and virtual source positions for electron beam.^4^ Historically, scan data are collected in liquid water since it provides a fluid medium for continuous movement of a radiation detector, and the fact it closely mimics radiation transport in the human body. In contrast, non‐scan data such as output factors may be measured in solid water even though solid water does not completely represent the properties of liquid water.^5^, ^6^ As a matter of convenience, solid water is commonly used for monthly quality assurance measurements due to ease of setup, whereas use of a water tank scan system is generally reserved for annual QA or commissioning when continuous scan data (or large sets of point measurements) are needed.

One issue with use of a water tank is that scans of large field sizes require long beam delivery times. Since the radiation detector effectively reports a point measurement, it must be stepped through the entire distance of the beam profile at a scan speed that maintains sufficient signal integration time to achieve low noise at each point. Detector arrays offer simultaneous point measurements at regularly spaced intervals, offering the potential to acquire scan data without the need to translate the system through the entire beam. Others have utilized detector arrays for leaf positioning accuracy and for the acquisition of commissioning measurements, but reported that the low data density limited its use for commissioning.^7^

Utilizing couch shifts with fractional distances of the detector spacing is one method to improve data density with a detector array. In this paper we demonstrate this method and use it to acquire variable resolution photon beam profiles and compare the data to commissioning scans acquired in a water tank.

## 2 MATERIALS AND METHODS

Scan profile data were acquired using an IC‐Profiler™ (Sun Nuclear, Melbourne, FL) and a TrueBeam™ linac (Varian, Palo Alto, CA). Translations of the detector array relative to the beam central axis (CAX) were used to acquire multiple measurements of a single radiation field size. To increase the data density a Python™ script interleaved data and a single aggregate scan profile was produced and renormalized. Data points were interleaved by accounting for the displacement from the CAX, and the final scan resolution exceeded the array detector spacing.

### 2.A Geometry and scan equipment

**Figure 1:**
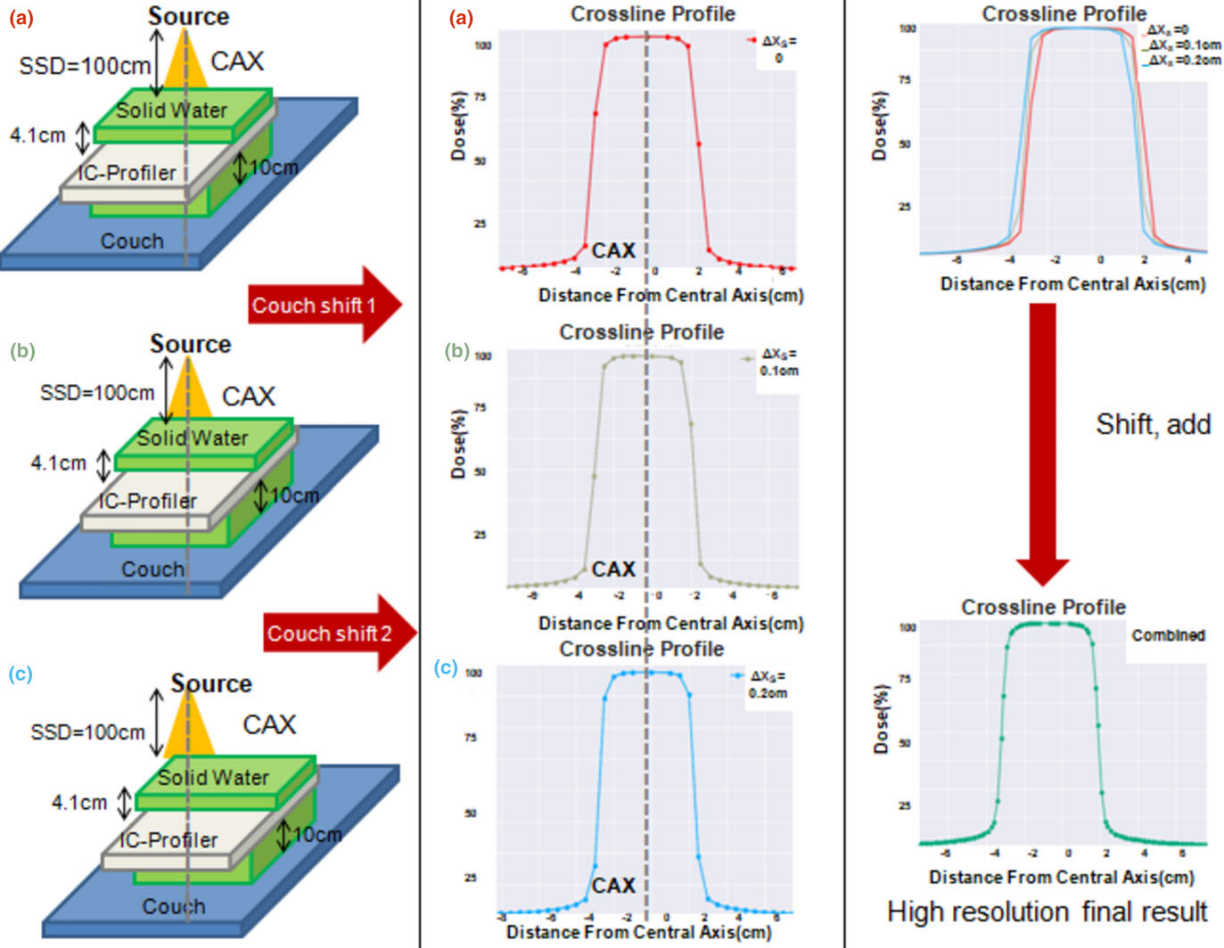
Experiment setup for 5 cm depth crossline acquired with couch shifts of (a) 0 cm, (b) 0.1 cm, and (c) 0.2 cm.

The IC‐Profiler™ is a multi‐axis ion chamber array with detector spacing along the *X* and *Y* axis of 5 mm, excluding the two detectors nearest to the center on *X* axis, and 7.07 mm on diagonal arrays. The measurement range along the *X* and *Y* axis is 32 and 45 cm on diagonals. The IC‐Profiler™ was placed on the couch atop 10.0 cm of solid water for backscatter and under varying thicknesses of solid water to achieve different depths (Fig. 1). The detector array was aligned with central axis (CAX) using the light field crosshairs, in‐room guidance lasers, and 50 monitor unit (MU) was delivered in the IC‐Profiler™ integration time. Field sizes and SSD values used for data acquisition are shown in Table 1. The *Y* detector array was used for the small field measurements because of the lack of the two detectors nearest to the center on *X* axis.

**Table 1:** Field sizes and SSD values used for data acquisition

Square field sizes (cm^2^)	Depths (cm)	SSD (cm)	MU	Energy (MV)	Solid water, square size (cm^2^)	Couch shift increment (cm)	Ic‐Profiler™ array axis
5	*d*_max_, 5, 10	100	50	6, 23	30	0.1	*X*‐Crossline
10	*d*_max_, 5, 10	100	50	6, 23	30	0.1	*X*‐Crossline
10	5 (EDW)	100	50	6	30	0.1	*X*‐Crossline
15	*d*_max_, 5, 10	100	50	6, 23	30	0.1	*X*‐Crossline
2	*d*_max_, 5, 10	100	50	6	30	0.05	*Y*‐Inline
25	5	100	50	6, 23	30	0.1	*X*‐Crossline
40	5	70	50	6	40	0.1	*X*‐Crossline

Due to the limited measurement range of the IC‐Profiler™ (32 cm in *X* and *Y*) relative to a 40 × 40 cm^2^ field size at 100 SSD, an SSD of 70 cm was used to geometrically scale the 40 × 40 cm^2^ field size down to 28 × 28 cm^2^. After acquisition of the SSD = 70 cm 40 × 40 cm^2^ field, the composite profile distance data were multiplied by the factor of 105/75 (i.e., considering the depth of the measurement which was 5 cm) for direct comparison to the water tank data that were acquired at 100 cm SSD. For full scatter conditions, 40 × 40 cm^2^ solid water slabs (CIRS, Norforlk, VA) were used for the 40 × 40 cm^2^ field size. For the smaller scan sizes, 30 × 30 cm^2^ solid water slabs (CIRS, Norforlk, VA) were used.

### 2.B Couch shift QA

Before starting the IC‐Profiler™ measurements, couch position quality control tests were performed on the ExacTrac^®^ (Brainlab, Munich, Germany) couch in the positive lateral and longitudinal directions. To do this, an image of the iBEAM^®^ Indexing bar was acquired using MV imaging with the electronic imaging portable device (EPID). The couch was shifted in increments of 0.01 cm and imaged between each subsequent shift. Next, the couch was shifted in increments of 0.1 cm and 1.0 cm with subsequent images acquired. The position of the iBEAM^®^ indexing bar relative to CAX was measured in the images for each shift.

### 2.C Scan reconstruction

All composite scan data was reconstructed using a custom Python™ software tool. To construct in‐line and crossline beam profiles from the detector array data, the IEC61217 coordinate system was used to transform from the IC‐Profiler™ coordinate system to the radiation isocenter coordinate system.^8^ In the *X* and *Y* directions, the 33rd detector was the central detector. The IC‐Profiler™ omits two detectors around the central detector in X direction. Equations (1) and 2 assign the position coordinates in units of cm. Since the array is 2D, there is only one detector in *Z* the direction.

(1) $$X_{P}=0.5*\left(X\;Detector\;number-33\right)$$

(2) $$Y_{P}=0.5*\left(Y\;Detector\;number-33\right)$$

**Figure 2:**
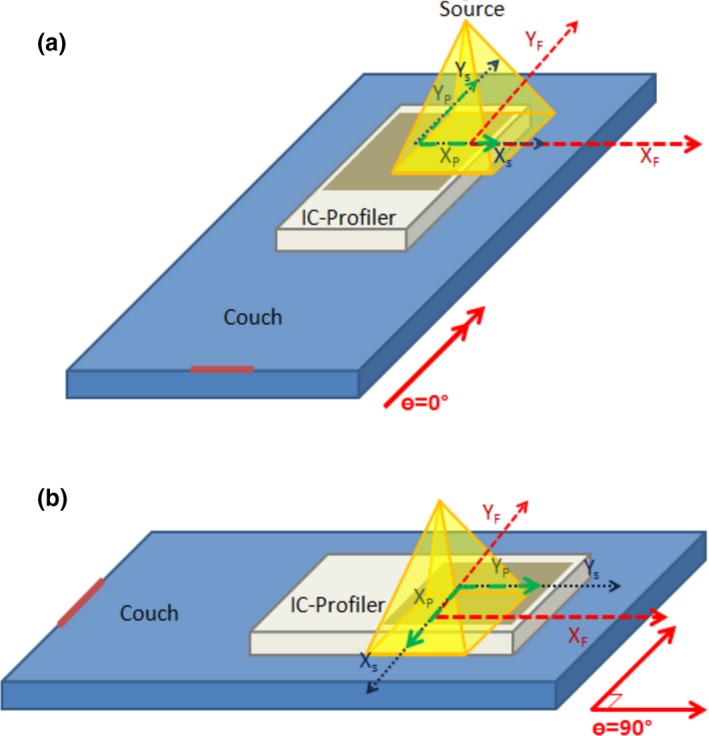
(a) Couch oriented normally (0°) and (b) Couch rotated 90° (agrees with IEC 61217 coordinate system).

Table 2 shows sample calculation of the coordinates based on the measurements. *X*_S_ and *Y*_S_ are the couch lateral and longitudinal position coordinates, respectively. Δ*X*_S_ and Δ*Y*_S_ are the differences between the successive values after applying a couch shift. If we consider that the radiation isocenter is located at (*X*_F_ = 0 cm, *Y*_F_ = 0 cm, Z_F_ = 0 cm), and the IC‐Profiler™ central detector is at (*X*_p_, *Y*_P_, *Z*_P_), after shifting the support (couch) to *X*_S_ = 0.1 cm, the IC‐Profiler™ will be located at (−0.1, 0, 0 cm) with respect to the isocenter. Equation (3) and (4) transform the IC‐Profiler™ data to the radiation isocenter coordinate system. This transformation is valid even if the couch is rotated 90°. Figure 2 illustrates the three independent coordinate systems when the couch is oriented normally (at 0°) and when it is rotated 90°.

**Table 2:** Sample calculation of the coordinates based on the measurements when the couch is oriented normally

Measurement number	*X*s initial (cm)	*X*s (cm)	Δ*X*s, shift (cm)	*X*_P_ (cm)	*Y*s initial (cm)	*Y*s (cm)	Δ*Y*s, shift (cm)	*Y*_P_ (cm)	Couch/support angle (deg)
1	0	0	0	0	147.5	147.5	0	0	0
2	0	0.1	0.1	−0.1	147.5	147.5	0	0	0
3	0	0.2	0.2	−0.2	147.5	147.5	0	0	0
4	0	0.3	0.3	−0.3	147.5	147.5	0	0	0
5	0	0.4	0.4	−0.4	147.5	147.5	0	0	0
6	0	0	0	0	147.5	147.5	0	0	0
7	0	0	0	0	147.5	147.6	0.1	−0.1	0
8	0	0	0	0	147.5	147.7	0.2	−0.2	0
9	0	0	0	0	147.5	147.8	0.3	−0.3	0
10	0	0	0	0	147.5	147.9	0.4	−0.4	0

(3) $$X_{F}=\left(X_{p}+\Delta X_{s}\right)\cos\left(\theta \right)+\left(Y_{p}+\Delta Y_{s}\right)\sin\left(\theta \right)$$

(4) $$Y_{F}=\left(Y_{p}+\Delta Y_{s}\right)\cos\left(\theta \right)+\left(X_{p}+\Delta X_{s}\right)\sin\left(\theta \right)$$

In the above equations: *X*_F_ = Fixed “*x*” position, *Y*_F_ = Fixed “*y*” position, *X*_P_ = IC‐Profiler™ “*x*” position, *Y*_P_ = IC‐Profiler™ “*y*” position, Δ*X*_S_ = Support (couch) “*x*” position, Δ*Y*_S_ = Support (couch) “*y*” position, and θ is the support (couch) rotation, as defined in Fig. 2.

To evaluate the effect of the reconstructed composite profile resolution on the gamma pass rate, scans were acquired and reconstructed with varying couch shift increments (0.50, 0.20, 0.15, and 0.10 cm shift increments). The field size used was 2 × 2 cm^2^ at 100 cm SSD and the depth of the measurement was 5 cm. Gamma analysis comparison to water tank data was performed at 2%/2 mm criteria.

### 2.D Gamma analysis comparison to water tank data

Gamma analysis was used to quantify the agreement between the IC‐Profiler™, and water tank profiles at the equivalent depth and field sizes using two gamma tolerance levels (1%/1 mm, and 2%/2 mm criteria).^9^ In order to calculate the equivalent depth, we assumed 5 cm solid water was same as 5 cm water in tank; also, we corrected for the IC‐Profiler™ shift to the effective point of measurement and the 0.9 cm inherent buildup in the IC‐Profiler™ array. The 3D Scanner water tank data were acquired during linac commissioning using the SNC125c (Sun Nuclear, Melbourne, FL) ion chamber for 5 × 5, 10 × 10, 15 × 15, 25 × 25, and 40 × 40 cm^2^ field sizes. The width of the ion chamber was oriented in the scan direction (smallest direction), and the measurement step size was 0.05 cm. The EDGE Detector™ (Sun Nuclear, Melbourne, FL) was used for 2 × 2 cm^2^ field size for both 6 and 23 MV photon beams. The SNC125c 0.125 cc volume ion chamber was used to measure fields equal and larger to 4 × 4 cm^2^. For the 2 × 2 cm^2^ field sizes the EDGE Detector™ was used to mitigate blurring in the penumbra regions.^4^ Gamma analysis was performed using the npgamma Python™ code.^10^ For fair comparison of the IC‐Profiler™ data and the EDGE Detector™ data, the EDGE detector data was convolved with a rectangular function whose width was that of the individual IC‐Profiler™ chamber.

### 2.E EDW

60° dynamic wedge (Varian EDW) scans at 6 and 23 MV photon energy were acquired for a 10 × 10 cm^2^ field size at 5 cm depth and 0.1 cm resolution. The scan data were compared to Eclipse™ (Varian, Palo Alto, CA) treatment planning system (TPS) exported data with a voxel size of 0.12 cm since the commissioning scans of enhanced dynamic wedge (EDW) fields were not available.

### 2.F Uncertainty

Type A and B uncertainties^11^ associated with the IC‐Profiler™ were calculated. The type A uncertainties for this study were due to setup and beam output variation, related to measuring the raw data for each detector on the x axis of the IC‐Profiler™ for five times while the setup was 23 MV, 15 × 15 cm^2^, at *d*_max_ = 3.6 cm. Type B uncertainty was due to the systematic uncertainties, e.g., couch position and detector array calibration. We recommend performing array calibration of the IC‐Profiler™ on a commissioned linac and by following the manufacturer recommendations.

## 3 RESULTS

### 3.A Scan reconstruction

**Figure 3:**
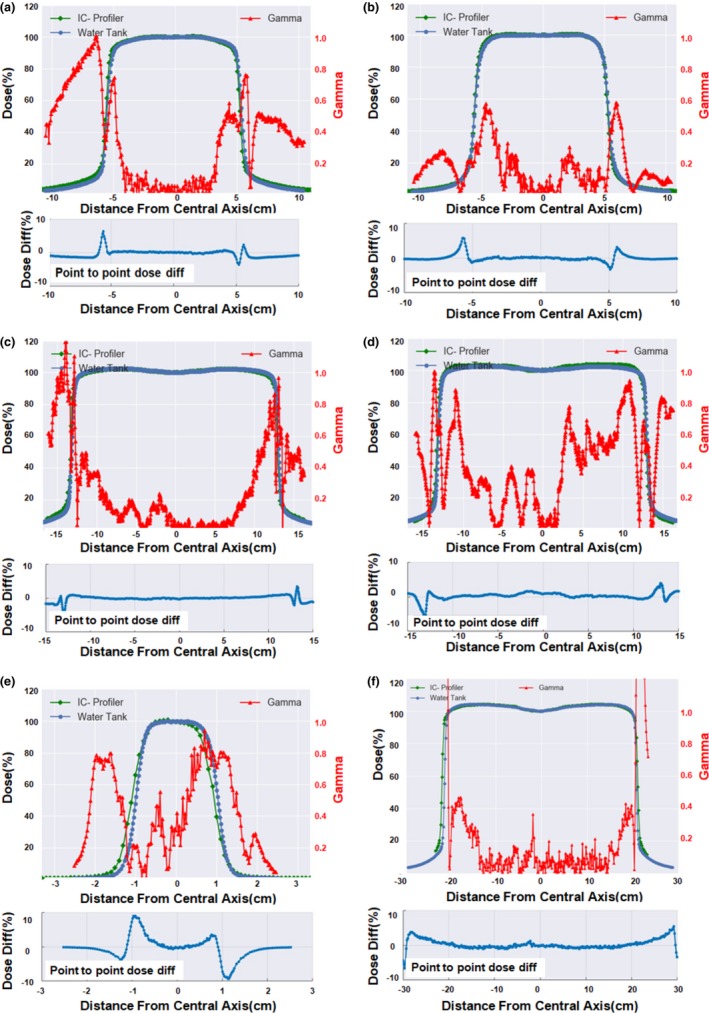
Graph of photon beam profile data at 2%/2 mm criteria. (a) 6 MV, 10 × 10 cm^2^ FS, 10 cm depth, gamma pass rate = 99.40% (b) 23 MV, 10 × 10 cm^2^ FS, 10 cm depth, gamma pass rate = 100% (c) 6 MV, 25 × 25 cm^2^ FS, 5 cm depth, gamma pass rate = 88.30% (d) 23 MV, 25 × 25 cm^2^ FS, 5 cm depth, gamma pass rate = 89.37% (e) 6 MV, 2 × 2 cm^2^ FS, 1.5 cm depth, gamma pass rate = 99.20% (f) 6 MV, 40 × 40 cm^2^ FS, 5 cm depth, gamma pass rate = 78.60%.

The custom Python™ script returned composite resolution scan profiles with point spacing 0.1 cm for the 5 × 5, 10 × 10, 15 × 15, and 25 × 25 cm^2^ field sizes, and a composite resolution scan with 0.05 cm point spacing for 2 × 2 cm^2^ field size. Figure 3 shows a subset of the IC‐Profiler™ data plotted alongside the water tank profiles used for commissioning. Point‐by‐point dose differences are displayed on an axis underneath the main profile for the profiles to show the accuracy of the IC‐Profiler™ data as a function of off‐axis distance. Good overall agreement was seen in the quantitative data represented as point‐by‐point gamma analysis values. An apparent lateral misalignment is unveiled between the two profiles for *E* = 6MV, depth = *d*_max_, field size = 2 × 2 cm^2^ [Fig. 3(e)]. For small fields, alignment of the ion chamber is more difficult due to the steep gradient of the fields. Additionally, a 0.2 mm error in the alignment will be more apparent in the plotted data due to the scale of the distance axes displayed.

### 3.B Gamma analysis comparison to water tank data

Table 3 shows the percentage of points satisfying gamma with 1%/1 mm and 2%/2 mm criteria for 6 and 23 MV photons, for different field sizes, and depths. It can be seen that most of the measurements had 100% gamma function pass rate at 2%/2 mm criteria. The lowest percentage of points satisfying gamma at 2%/2 mm criteria was 76.13% for 6 MV, 40 × 40 cm^2^ field size at 5 cm depth. At 1%/1 mm the highest gamma function pass rate was 99.38% for 6MV, 10 × 10 cm^2^, at *d*_max_, and the lowest at this criterion was 51.5% for 6 MV, 2 × 2 cm^2^ field size at *d*_max_.

**Table 3:** Percentage of points satisfying gamma with 1%/1 mm and 2%/2 mm criteria scans. “–” indicates data were not collected. The data for 2 × 2 cm^2^ are after convolution

Square field size (cm^2^)	Depth (cm)	Shift (cm)	(2%/2 mm) gamma pass rate		(1%/1 mm) gamma pass rate	
			6 MV	23 MV	6 MV	23 MV
2	1.5	0.05	94.2	–	51.5	–
	5	0.05	100	–	65.7	–
	10	0.05	100	–	63.4	–
5	1.5	0.1	100	–	96.0	–
	3.6	0.1	–	100	–	79.9
	5	0.1	100	100	90.6	78.9
	10	0.1	99.4	100	97.8	79.3
10	1.5	0.1	100	–	99.38	–
	3.6	0.1	–	100	–	95.7
	5	0.1	100	100	87.5	95.1
	10	0.1	99.4	100	69.9	95.8
15	1.5	0.1	100	–	97.77	–
	3.6	0.1	–	100	–	96.08
	5	0.1	99.30	100	75.48	90.78
	10	0.1	95.99	100	66.5	96.42
25	5	0.1	88.30	89.37	72.85	51.79
40 (SSD = 70 cm)	5	0.1	76.13	–	63.44	–

**Figure 4:**
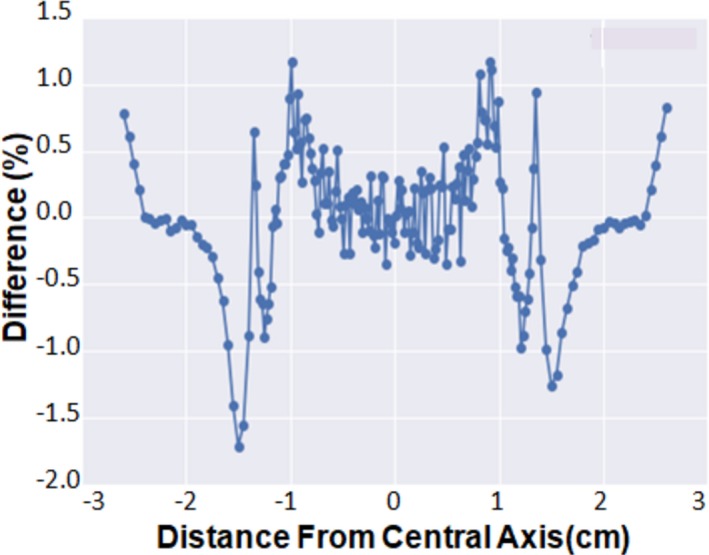
Dose difference between the convolution and non‐convolution method for 6 MV, 2 × 2 cm^2^ field size, 10 cm depth.

Table 4 summarizes the convolved EDGE Detector™ data comparison to the IC‐Profiler™ data at the 2 × 2 cm^2^ field size. Good agreement to within about 95% pass rate was seen at the 2%/2 mm level. Note, without convolution, the pass rate was drastically reduced to only 60%. This reduced pass rate suggests that the IC‐Profiler™ can measure small field sizes at 2 × 2 cm^2^, but the resultant profiles are blurred by the detector size due to the volumetric effects. Figure 4 plots dose difference between the convolution and non‐convolution method for 6 MV, 2 × 2 cm^2^ field size, and 10 cm depth. The difference between these two is minor in all regions with the exception of the field edges. Lack of agreement is expected at the edges due to the high gradient regions and the resultant volumetric averaging effect of the larger detector volume in the IC‐Profiler™.

**Table 4:** Dosimetric agreement for small field 3D Scanner tank data (Edge detector) convolved to match measured IC‐Profiler™ data. *E* = 6 MV, inline scans, Composite Point Spacing = 0.05 cm

Square field size (cm^2^)	Depth (cm)	(1%/1 mm) gamma pass rate		(2%/2 mm) gamma PASS rate	
		No‐convolution (%)	Convolution (%)	No‐convolution (%)	Convolution (%)
2	1.5	51.1	51.5	86.5	94.2
2	5	65.0	65.7	99.4	100
2	10	59.7	63.4	99.5	100

The gamma pass rate of the 40 × 40 cm^2^ field size for the water tank (100 SSD) and IC‐Profiler™ (70 SSD) was 76.13% at 2%/2 mm. Therefore, while technically possible to capture the field at a reduced SSD, the data shows large discrepancy in the gamma pass rate. Data acquisition at reduced SSD is thus not recommended by the authors, which limits the maximum field size that can be measured to 32 cm in *X* or *Y*.

Data in Table 5 shows gamma pass rate to increase as the resolution increases, i.e., more couch shifts yield better agreement to the commissioning water tank data. At the highest resolution of 0.05 cm the gamma pass rate was 100% using 2% 2 mm criteria, and 65.75% using 1% 1 mm criteria.

**Table 5:** Dosimetric agreement of 2 × 2 cm^2^, 3D Scanner tank data (Edge detector) convolved to match IC‐Profiler™ data at various composite point spacing. *E* = 6 MV, inline scans, 5 cm depth

Composite point spacing (cm)	Sampled points increase factor	Gamma pass rate (%)	
		1%/1 mm	2%/2 mm
0.50 (no shifts)	1.0×	22.6	34.3
0.20	2.5×	40.3	70.2
0.15	3.3×	58.6	75.1
0.10	5.0×	62.4	91.2
0.05	10.0×	65.7	100

### 3.C EDW

**Figure 5:**
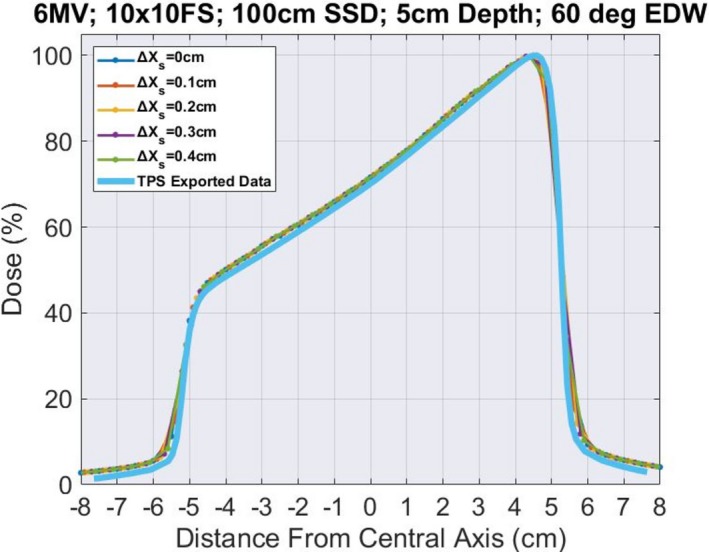
Comparison between 60° EDW for 6 MV, 100 cm SSD, 5 cm depth, 10 × 10 cm^2^ field size using IC‐Profiler™ composite (composite point spacing = 0.1 cm) and TPS Exported Data with Y2 jaw, and voxel size 0.12 cm. Normalization was only performed on the composite profile.

Figure 5 shows the 60° EDW profile for 10 × 10 cm^2^ field size for 6 MV with composite point spacing 0.1 cm, and TPS exported data with voxel size 0.12 cm. The measured data from the IC‐Profiler™ were combined in Python and the dose for each point was subtracted from the corresponding dose of the water tank data. The average of these subtractions was 3.02%.

### 3.D Uncertainty

Type A uncertainty was due to the change in the output of each detector from run to run. To calculate type A error, five measurements were performed for the 23 MV, 50 MU photon beam, 15 × 15 cm^2^ at *d*_max_ = 3.6 cm. The standard deviation of these measurements was calculated for each detector on the X array of IC‐Profiler™ (overall 63 detectors). The average of the output of each detector was also calculated for these five measurements. Coefficient of variation (COV) which is standard deviation divided by average was calculated. The average of COV in the infield region was 0.03%, a value that indicates a low value of Type A error and good repeatability between subsequent measurements.

One of the sources of the Type B uncertainty was due to couch positioning. Before taking measurement, an EPID image was taken from an iBEAM^®^ indexing bar. The couch was shifted laterally 0.4 cm, in increments 0.1 cm to the right and left, and each time the distance between the center of the image and right edge of the iBEAM^®^ indexing bar image was measured. The same procedure was performed in the longitudinal direction. Next the difference between adjacent distance from the right edge of the iBEAM^®^ indexing bar to the center of the image and adjacent couch shifts were measured and averaged. The average of the measured values was 0 ± 0.02 cm in both lateral and longitudinal direction which is within the tolerance. Detector array calibration was another source of the Type B uncertainty. The water tank scan data were misaligned by 0.02 cm.

## 4 DISCUSSION

Within the range of field sizes from 5 × 5 to 15 × 15 cm^2^, the IC‐Profiler™ array was able to acquire profile scans of 6 and 23 MV beams within 2%/2 mm agreement to a water tank scanning system. Note that a correction factor was not applied for the comparison of solid water and liquid water measurements. 1%/1 mm agreement was substantially lower, suggesting that this technique can not accurately reconstruct measured profiles to better than 2%/2 mm. While the demanding gamma criterion in patient QA is 2%/2 mm, a DTA of 2 mm (may imply a deviation of 10% of the field size for a (2 × 2 cm^2^) field. The reason for not measuring 23 MV for the small fields (2 × 2 cm^2^) is that we don't do small field treatments with high energy (23 MV). Also, clinically we don't use 40 × 40 field sizes for high energy (23 MV).

Dose profiles agreed best to the water tank data in regions where the dose >20% of *D*_max_. However, the gamma pass rates decreased in the tail regions (primarily regions of scatter), likely due to differences in the geometrical construction of the IC‐Profiler™ detector compared to the homogenous geometry of a water tank. These geometrical factors, such as the high density of air pockets (each chamber) and the use of high‐Z materials, result in material interface effects and perturbation of the scatter components.

This method may be best suited to supplement water tank scans of large field sizes; within the range of field sizes that can be acquired with the IC‐Profiler™ (32 cm in *X* and *Y* at 100 cm SSD). To acquire a 15 × 15 cm^2^ scan, this method used 250 MU (0.1 cm composite point spacing) vs 1040 MU for a commissioning scan continuously acquired in a 3D water tank, representing a fourfold increase in efficiency in the beam on‐time. There is a trade‐off between beam on time (more MU more on time) and the measurement uncertainty (less MU more uncertainty in a single IC‐Profiler™ reading). 50 MU was selected because it achieved a good compromise between the beam on time and resulted in negligible differences in the ion chamber reading between subsequent measurements of 0.03% standard deviation for a single ion chamber reading in the field. MU deliveries of 10, 50, and 100 MU were tried before selecting the 50 MU). Also, it is not possible to refine the spatial resolution of an array (with given intrinsic spatial resolution of the detectors) endlessly by decreasing the scanning step.

Since high‐z material is used in the construction of the IC‐Profiler™, it is not recommended by the authors to use IC‐Profiler™ for the depth dose measurement due to the beam hardening artifacts. Also, the purpose of this research is to acquire data efficiently from outside of the room, but performing the depth dose measurement requires the physicist to go in the room for each measurement and move the water to create a new depth.

Acquisition of 40 × 40 cm^2^ field size at reduced SSD of 70 cm to measure the full profile did not show good agreement with water tank 40 × 40 cm^2^ profile. This suggests that the IC‐Profiler™ should only be used up to the maximum 32 cm field size, and always at 100 cm SSD. Additionally, the authors do not recommend use of this technique for small field sizes <4 × 4 cm^2^ at 1%/1 mm criteria, since the average of the percentage of points satisfying gamma in different depths was 60.2%.

## 5 CONCLUSION

Reconstructed scans from an ion chamber profile array were achieved for photon beam profiles at 0.05 cm point spacing for 2 × 2 cm^2^ field size and 0.1 cm point spacing for 5 × 5, 10 × 10, 15 × 15, 25 × 25, and 40 × 40 cm^2^ field sizes. Reasonable agreement to water tank ion chamber measurements was seen with 2%/2 mm criteria for field sizes in the range of 5 × 5 to 25 × 25 cm^2^ This technique opens up new possibilities for rapid acquisition of variable resolution, 2D–3D dosimetric data for QA checks of baseline data (which were acquired with a tank).The IC‐Profiler™ agreement did not achieve >90% pass rate at 1%/1 mm tolerance as compared to 3D water tank data, suggesting that a water tank scan system is still required and it is not suggested to replace the need to measure profiles in the tank at the time of commissioning. For field sizes larger than 15 × 15 cm^2^, this method decreased the total number of MU needed by more than a factor of four, while maintaining comparable data quality to a water tank scan system within 2%/2 mm. This technique may be coupled with scripted couch movements and automated beam delivery (e.g., via Varian Developer Mode) to further improve on efficiency.

## CONFLICT OF INTEREST

The authors declare no conflict of interest.
